# Comparison of four staining methods for detecting eosinophils in nasal polyps

**DOI:** 10.1038/s41598-018-36102-y

**Published:** 2018-12-07

**Authors:** Yu Song, Jinshu Yin, Hong Chang, Quan Zhou, Hong Peng, Wei Ji, Qingkun Song

**Affiliations:** 1 Peking University Ninth School of Clinical Medicine, Beijing, 100038 China; 2 Department of Otorhinolaryngology-Head and Neck Surgery, Beijing Shijitan Hospital of Capital Medical University, Beijing, 100038 China; 3 Department of Pathology, Beijing Shijitan Hospital of Capital Medical University, Beijing, 100038 China; 4 Department of science and technology, Beijing Shijitan Hospital of Capital Medical University, Beijing, 100038 China

## Abstract

The study aimed to find a more appropriate method to detect eosinophils in formalin- fixed nasal polyps, since there is no consensus on the standard counting method of eosinophils now. Four 5 μm serial sections were obtained from each 10% neutral formalin-fixed paraffin block and were stained with Chromotrope 2R, Congo red, MBPmAb immunohistochemistry, and conventional hematoxylin and eosin stain respectively. Each section was scanned by the Aperio digital section scanner. The same selected areas were procured for assessment in the serial sections. Chromotrope 2R and MBPmAb immunohistochemistry were specific in detecting eosinophils, which had the lower background staining compared with Congo red and conventional hematoxylin and eosin stain. There were significant differences among the four methods in terms of the eosinophil counting data (*p* < 0.05), while no significant difference between Chromotrope 2R and Congo red (*P* = 0.1413). The eosinophil counts in nasal polyps could be more accurately assessed by Chromotrope 2R and Congo red compared with MBPmAb immunohistochemistry and conventional hematoxylin and eosin stain. The popularization of Chromotrope 2R and Congo red may help to unify the eosinophil count in the definition of eosinophilic CRSwNP.

## Introduction

Chronic rhinosinusitis with nasal polyps (CRSwNP), a multifactorial and highly heterogeneous upper airway disease, is a severe phenotype of chronic rhinosinusitis and presents with distinct immunological and histopathological features compared with chronic rhinosinusitis without nasal polyps (CRSsNP)^1,2^. Clinically, CRSwNP is classified into two phenotypes based on the dominant inflammatory cell type: eosinophilic CRSwNP and non-eosinophilic CRSwNP^3,4^. It is demonstrated that the degree of eosinophil infiltration in nasal polyps has a significant impact on their clinical characteristics, surgical timing, drug efficacy, and prognosis^5,6^.

There are no standard methods for eosinophils counting in nasal polyps now. Conventional hematoxylin and eosin stain (HE) staining is used to perform manual counting based on the morphological features of eosinophils. However, it may become difficult since the eosin dye can stain the cytoplasm of all cells to different degrees of red, when the cell morphology is not typical or intensive infiltration with other inflammatory cells, especially neutrophil infiltration. We try to make sure if Chromotrope 2R^7^, Congo red^8^ and Immunohistochemistry^9^ could be used in nasal polyps to count eosinophils accurately. Thus, we performed this study aiming to compare the four methods mentioned above to find out the appropriate method to count eosinophils for distinguishment between eosinophilic CRSwNP and non-eosinophilic CRSwNP.

## Materials and Methods

This study was approved by the ethics committee of Beijing Shijitan Hospital of Capital Medical University and written informed consent was obtained from each patient. All methods were performed in accordance with the relevant guidelines and regulations. From June 2016 to June 2017, 27 patients with CRSwNP from Beijing Shijitan Hospital of Capital Medical University were selected for the study. The patients were diagnosed of CRSwNP based on European Position Paper on Rhinosinusitis and Nasal Polyps 2012 guidelines^1^. Patients with diagnosis of classic allergic fungal sinusitis, unilateral lesions, allergic fungal sinusitis, posterior nasal polyps, and sinus cysts were excluded in the study.

Four 5 μm serial sections were obtained from each 10% neutral formalin-fixed paraffin block. All sections were dewaxed and hydrated through graded alcohols and dipped in water, and then were stained with conventional HE, Chromotrope 2R, Congo red and immunohistochemistry respectively.

For conventional HE staining, all slides were stained at Department of Pathology of Beijing Shijitan Hospital following a widely used protocol described elsewhere^10^.

The staining protocol of Chromotrope 2R was performed as previously described^7,11^. Sections were immersed in hematoxylin for 5 minutes at 25 °C before being rinsed in running tap water, then stained with 0.5% Chromotrope 2R solution for 30 minutes at 25 °C. 0.5% Chromotrope 2R solution were prepared by adding 1 g of phenol (P5566, Sigma-Aldrich) to 100 ml of gentle heat water followed by 0.5 g of Chromotrope 2R (C3143, Sigma-Aldrich).

The protocol of Congo red was slightly modified according to the previous description to optimize eosinophil detection^8,12^. Sections were immersed in Hematoxylin for 5 minutes at 25 °C before being rinsed in running tap water, then stained with 0.5% alcoholic Congo red solution (C6277, Sigma-Aldrich) for 15 minutes (reduced from the original 20–30 minutes to 15) at 25 °C.Then, the sections were placed into 75% alcohol solution to differentiate for a few seconds.

After dewaxed in xylene for 10 minutes three times and rehydration, antigen was retrieved 0.4% pepsin for 15 minutes at 37 °C. Peroxidase activity was inactivated using 3% hydrogen peroxide solution for 5 minutes at room temperature. Subsequently, the sections were incubated with mouse anti-human eosinophil major basic protein (MBP) monoclonal antibody (BMK13, Bio-Rad) at a 1:20 dilution overnight at 4 °C^13,14^. The next day, after washing with aqueous buffer, the goat anti-mouse secondary antibody IgG conjugated with horseradish peroxidase (PV-8000, ZSGB-BIO) was added and incubated for 25 minutes at 37 °C. 3,3-Diaminobenzidine was added to stain the slide at 25 °Cand all specimens were counterstained with hematoxylin. Sections incubated with only the secondary antisera were used for negative controls.

### Qualitative assessment

Qualitative assessments of staining parameters were performed by experienced pathologists, including specificity of eosinophil staining and background staining.

### Quantitative assessment and statistics

**Figure 1:**
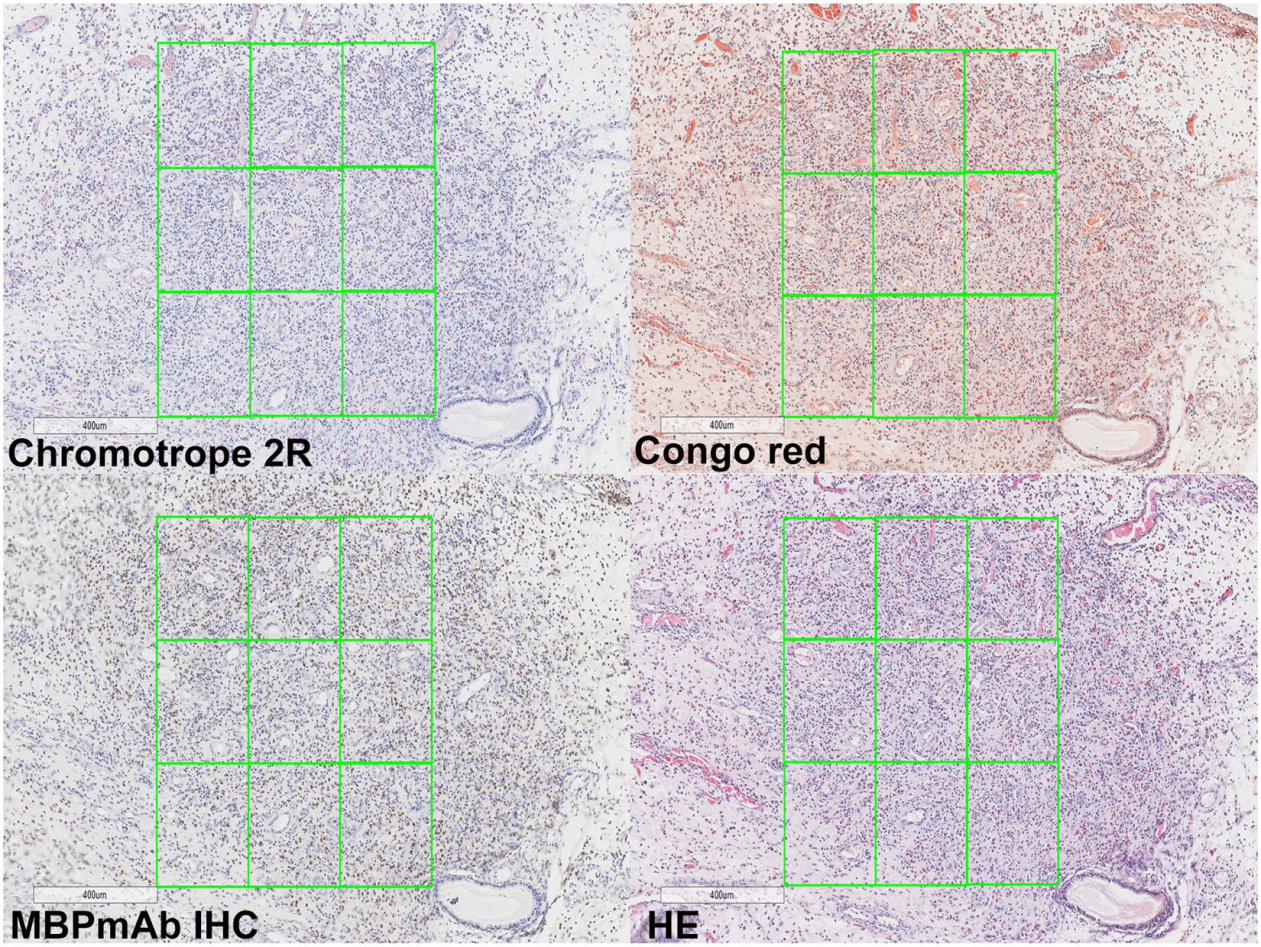
Select the same area for quantitative assessment (×50).

All stained sections were scanned by the Aperio AT Turbo (Leica Microsystems). ImageScope 12.1(Leica Microsystems) was used to select 9 consecutive square areas for each case. The area of each small square area was fixed to 0.09 mm^2^ and the same selected areas were procured for assessment in the serial sections (Fig. 1). Counting eosinophil was performed by experienced pathologists.

All of the statistical analyses were performed using the GraphPad prism 5. The counting data were not normally distributed showed by the Shapiro-Wilk’s test. Therefore, the Friedman test was performed to determine whether there were significant differences among the conventional HE, Chromotrope 2R, Congo red and MBPmAb IHC for the eosinophil counting data. The Wilcoxon signed-rank test was used for the pairwise comparisons, if the significant differences were noted. The Bonferroni correction was applied in pairwise comparisons and the adjusted *p* = 0.05/6 was considered significant. All tests were done using the two-tailed option. For all analyses, *P* < 0.05 was considered significant.

## Results

### Qualitative assessment

**Figure 2:**
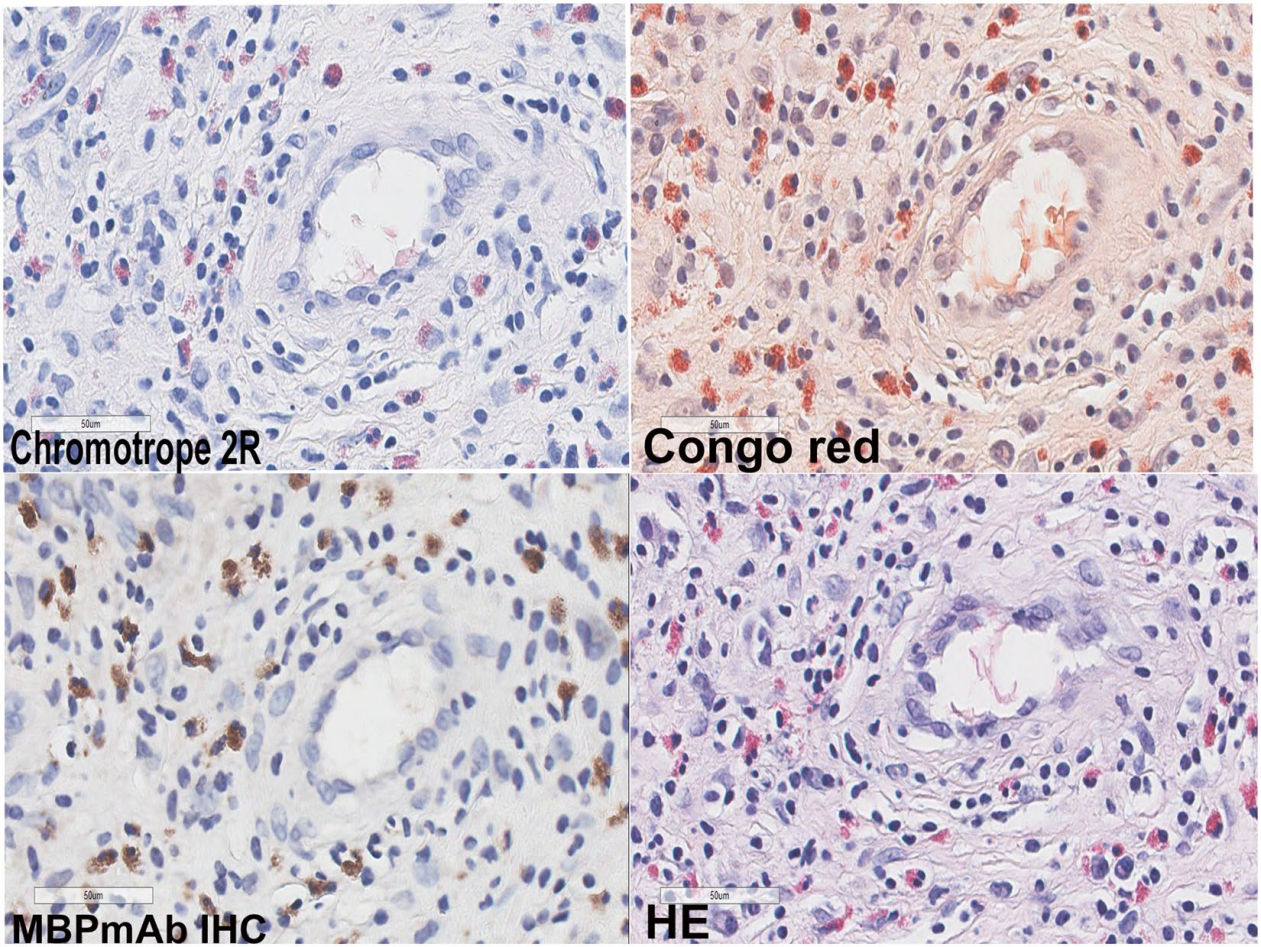
Background staining among the four methods. (×400).

For Chromotrope 2R, Congo red and MBPmAb IHC, none of them stained neutrophils, lymphocytes, plasma cells or mast cells, indicating that these stains specifically stain eosinophils. For conventional HE, the overlapping staining pattern and morphology of eosinophils versus neutrophils sometimes may be the problem. Figure 2 showed the background staining among Chromotrope 2R, Congo red, MBPmAb IHC and conventional HE. It was clear that Chromotrope 2R2R and MBPmAb IHC showed less background staining compared with Congo red and conventional HE.

### Quantitative assessment and statistics

**Figure 3:**
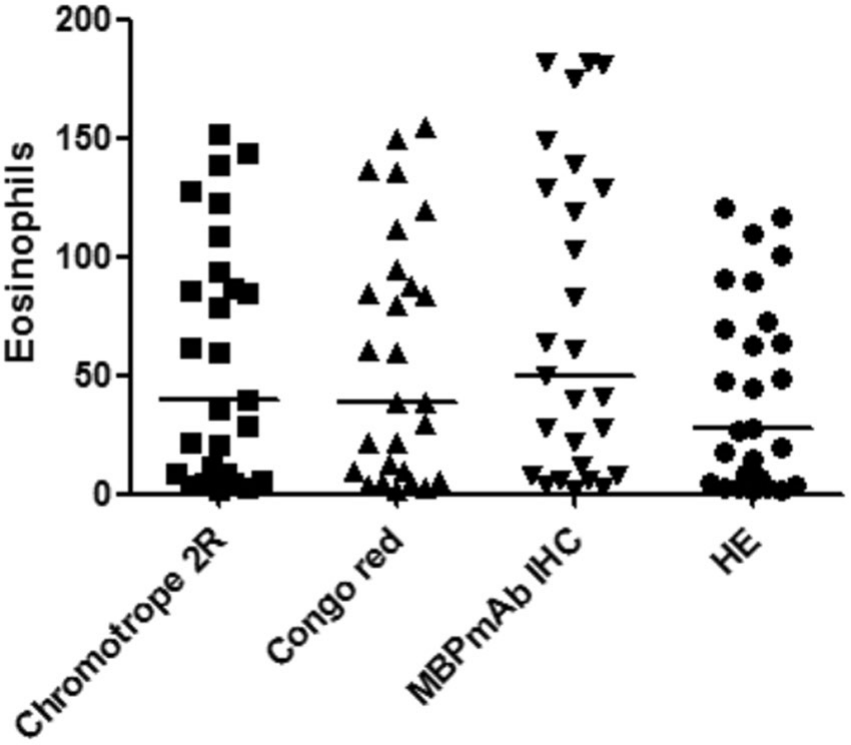
Eosinophil counting data among the four staining methods.

There were significant differences among the four staining methods (*p* < 0.05).The conventional HE method yielded a lower eosinophil counting than Chromotrope 2R (*p* < 0.05), MBPmAb IHC (*p* < 0.05),and Congo Red (*p* < 0.05),while the MBPmAb IHC yielded a higher eosinophil counting than Chromotrope 2R (*p* < 0.05), conventional HE (*p* < 0.05),and Congo Red (*p* < 0.05).Interestingly, there was no significant difference between Congo red staining and Chromotrope 2R staining (*P* = 0.1413). (Figure 3).

## Discussion

Compared with non-eosinophilic CRSwNP, eosinophilic CRSwNP is quite different in terms of clinical symptoms, treatment, and prognosis. Generally, patients with eosinophilic CRSwNP are more prone to have nasal congestion, sneezing, and olfactory dysfunction; they are more sensitive to local or systemic steroids than macrolides; Besides, they are more likely to have recurrence after surgery. However, macrolides have greater effect than local or systemic steroids for patients with eosinophilic CRSwNP^15^. Therefore, it’s important to distinguish the two types accurately and provide individualized treatments.

The standard eosinophil count in the definition of eosinophilic CRSwNP has not yet reached a consensus till now. Some researchers believe that the number of eosinophils in each high-power field (HPF) should be 10/HPF^16,17^, 20/HPF^18^, 50/HPF^19,20^, 70/HPF^4,21^ or 100/HPF^22^, while others confirm that it should be defined by the ratio of eosinophils to other inflammatory cells in the same field of vision^23^.

At present, conventional HE is widely used for counting eosinophils in nasal polyps as this method can reflect the general situation of eosinophil infiltration. However, the eosin can stain the cytoplasm of all cells to different degree of red non-specifically, therefore the cytoplasm of eosinophils are deep red due to the granulars while the neutrophils are stained light red. Consequently, it may become difficult to differentiate eosinophils from neutrophils and it’s easy to cause visual fatigue when reading numerous slides.

According to the results of this study, Chromotrope 2R staining is prominent in terms of specificity and background staining and seems to be a suitable staining method. However, When the tissue is over-fixed in 10% neutral formalin, it can affect the staining. In addition, gentle heat water was used to dissolve the phenol, because phenol was slightly soluble in water at room temperature, and it is miscible with water of any proportion above 65 °C.

Eosinophils can be dyed orange-red color clearly with Congo red, in distinct contrast with other cellular components. However, elastic fibers also be stained red if they are present in the nasal polyp samples, which increases background staining. In order to control the background staining of Congo red, we found it can be reduced by immersing the sample in 75% ethanol to differentiate, generally 30 seconds, after immersing it in 0.5% ethanol Congo red solution for 15 minutes.

**Figure 4:**
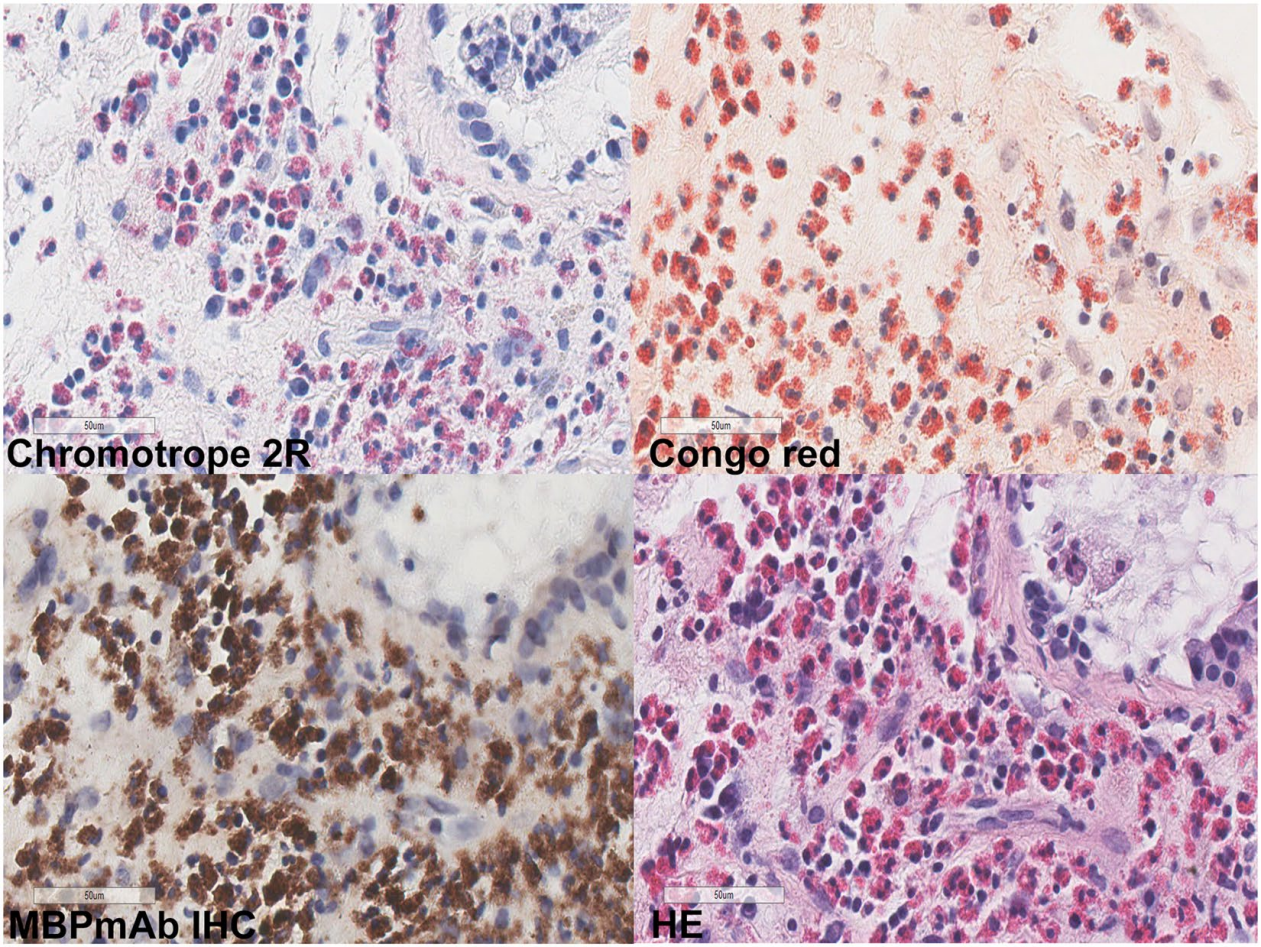
The staining of eosinophil dense area. (×400).

Although MBP serves as a marker of eosinophils and is used to observe the distribution and degranulation of eosinophils^24^, it may not be a suitable method for eosinophil counting. It is found that MBP-positive area may contaminate the nuclei and make it difficult to distinguish the number of cells. Although the eosinophils are connected together, the other histochemical positive signals do not completely cover the nucleus. In massive degranulation, immunohistochemistry may also stain peripheral cells, thus leading to higher counts (Fig. 4).

According to the study, both Chromotrope 2R and Congo red are specific and reproducible, and more suitable for accurate eosinophils counting in nasal polyps. The popularization of Congo red staining and Chromotrope 2R staining may help unify the eosinophil count in the definition of eosinophilic CRSwNP.
